# MACS: mild by definition, challenging by nature

**DOI:** 10.3389/fendo.2026.1957127

**Published:** 2026-10-26

**Authors:** Gabriela Bluszcz, Zuzanna Roszkowska, Jan Domański, Ahmad Sharabi, Joanna Rapiejko, Jerzy Małek, Urszula Ambroziak, Małgorzata Bobrowicz

**Affiliations:** 1 Department of Internal Medicine and Endocrinology, Medical University of Warsaw, Warsaw, Poland; 2 Doctoral School of the Medical University of Warsaw, Warsaw, Poland; 3 Student Scientific Club “Endocrinus” Affiliated to the Department of Internal Medicine and Endocrinology, Medical University of Warsaw, Warsaw, Poland

**Keywords:** adrenal incidentaloma, adrenalectomy, cardiometabolic risk, cortisol, frailty, mild autonomous cortisol secretion, steroidogenesis inhibitors

## Abstract

Mild autonomous cortisol secretion (MACS) is the most frequent form of cortisol excess in patients with adrenal incidentalomas. Although it lacks the classical phenotype of overt Cushing’s syndrome, MACS is increasingly recognized as a clinically relevant condition associated with multisystem complications rather than a benign biochemical finding. This narrative review summarizes current evidence on the pathophysiology, clinical manifestations, diagnostic limitations, and management of MACS, with particular focus on cardiometabolic, skeletal, muscular, neuropsychiatric, frailty-related, reproductive, and emerging microbiome-related consequences. Chronic exposure to low-grade cortisol excess is being recognized as a factor associated with hypertension, disorders of glucose metabolism, dyslipidemia, increased cardiovascular risk, metabolic dysfunction-associated steatotic liver disease, skeletal fragility, impaired muscle function, frailty, sleep and circadian disturbances, cognitive and mood alterations, hypogonadism, and possible changes in gut microbiota. The 1 mg overnight dexamethasone suppression test remains the cornerstone of diagnosis, but its interpretation is limited by assay variability, medication interference, comorbidities, and imperfect reflection of long-term tissue glucocorticoid exposure. Emerging approaches, including steroid profiling, hair cortisol/cortisone, salivary cortisone, inflammatory indices, metabolomic tools, and exploratory biomarkers related to microbial metabolites, may improve risk stratification in the future but remain investigational. Management should be individualized. Adrenalectomy may benefit selected patients with unilateral disease and clinically relevant cortisol-related comorbidities, whereas evidence supporting pharmacological therapy remains limited. MACS should be regarded as a heterogeneous multisystem condition requiring structured clinical assessment and individualized management. Prospective studies are needed to define clinically meaningful endpoints, refine diagnostic thresholds, and identify patients most likely to benefit from intervention.

## 1 Introduction

Mild autonomous cortisol secretion (MACS) is a state of adrenocorticotropic hormone (ACTH)-independent cortisol hypersecretion in patients with benign adrenal lesions, without clinical features of overt Cushing’s syndrome (CS) (1, 2). According to the 2023 European Society of Endocrinology (ESE) and European Network for the Study of Adrenal Tumours (ENSAT) guidelines, MACS should be considered when serum cortisol remains >50 nmol/L (>1.8 µg/dL) following a 1 mg dexamethasone suppression test (DST) in patients without signs and symptoms of overt CS (1). ACTH independence of cortisol secretion should be confirmed, typically by a suppressed or low-normal morning plasma ACTH concentration (1).

Adrenal incidentalomas (AIs) are detected in 1.4-7% of the adult population, with prevalence increasing with age and reaching up to 10% in people older than 70 years (3–5). The majority (80-96%) are benign (3) and 19-50% are associated with MACS (3). Differences across ethnic and demographic groups have been reported. In Western populations, incidentalomas are more frequently detected in women (58-64% of cases) at a mean age at diagnosis of 58–61 years (3, 6). However, for MACS specifically, the proportion of women is even higher (66–69%) and affected patients are older (median age 63–64 years) than those with non-functioning adrenal tumors (NFAT) (median age 58–60 years) (3). Patients with MACS typically present with larger adrenal masses and more frequent bilateral lesions (22-30% of cases) compared with patients with NFAT (3, 7).

The somatic mutational profile of adrenal adenomas associated with MACS appears to resemble that of NFAT more closely than that of adenomas causing overt Cushing’s syndrome (8, 9). MACS and NFAT are characterized by somatic catenin beta-1 (*CTNNB1*) mutations causing constitutive activation of the Wnt/β-catenin pathway (8–10). In contrast, activating mutations in protein kinase cAMP-activated catalytic subunit alpha (*PRKACA*) and GNAS complex locus (*GNAS*), which stimulate the cyclic adenosine monophosphate-protein kinase A (cAMP–PKA) pathway, are less common in MACS than in overt CS. In patients with bilateral adrenal lesions, MACS is frequently associated with primary bilateral macronodular adrenal hyperplasia and germline armadillo repeat containing 5 (*ARMC5*) mutations, which are associated with a more severe phenotype and additional findings, such as meningiomas, which may indicate the need for genetic testing (11, 12).

Although clinical progression from MACS to overt CS is extremely rare (<1% of cases) (1, 7), chronic cortisol hypersecretion has significant cardiometabolic consequences (**Figure 1**) (7, 13–17).

**Figure 1:**
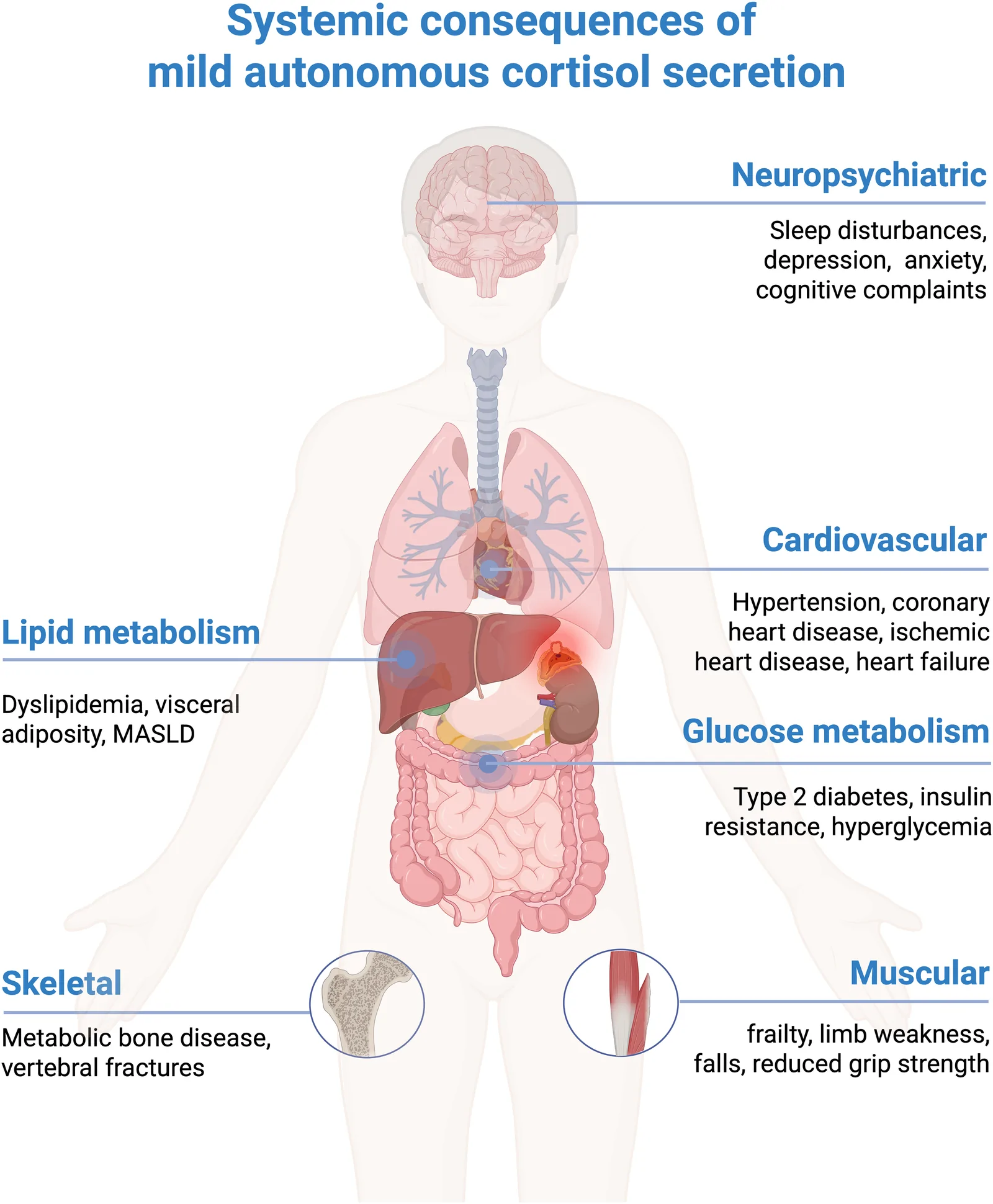
Systemic consequences of mild autonomous cortisol secretion, including cardiometabolic, skeletal, muscular, neuropsychiatric, and frailty-related associations. The figure shows the summary of reported associations; not all pathways have been demonstrated to be causal in MACS. MASLD, metabolic dysfunction-associated steatotic liver disease.

Because MACS lacks classical signs of overt hypercortisolism (**Figure 2**), its clinical presentation overlaps with metabolic syndrome, complicating the differential diagnosis (2, 18). Notably, the increased cardiovascular risk and mortality are independent of preexisting diabetes or hypertension (4). Although proposed DST cut-off values show poor overall accuracy in predicting comorbidities, studies indicate that progressively higher post-DST cortisol levels correlate with a greater cardiometabolic risk (1, 3). Beyond the degree of cortisol hypersecretion, cardiometabolic burden is influenced by individual genetic susceptibility, peripheral glucocorticoid metabolism (e.g., 11β-hydroxysteroid dehydrogenase activity), and tissue sensitivity to glucocorticoids (14, 18). A meta-analysis of 5,921 patients demonstrated that all-cause, comorbidity-adjusted mortality was substantially higher in MACS compared with NFAT (hazard ratio [HR]=1.54, 95% confidence interval [CI] 1.27-1.81) (19). Therefore, the clinical management of MACS necessitates a thorough evaluation of comorbidities. In this context, the aim of our review is to provide a comprehensive answer to the question of why MACS matters, highlighting its clinical relevance, impact on patient outcomes, and implications for diagnosis and management.

**Figure 2:**
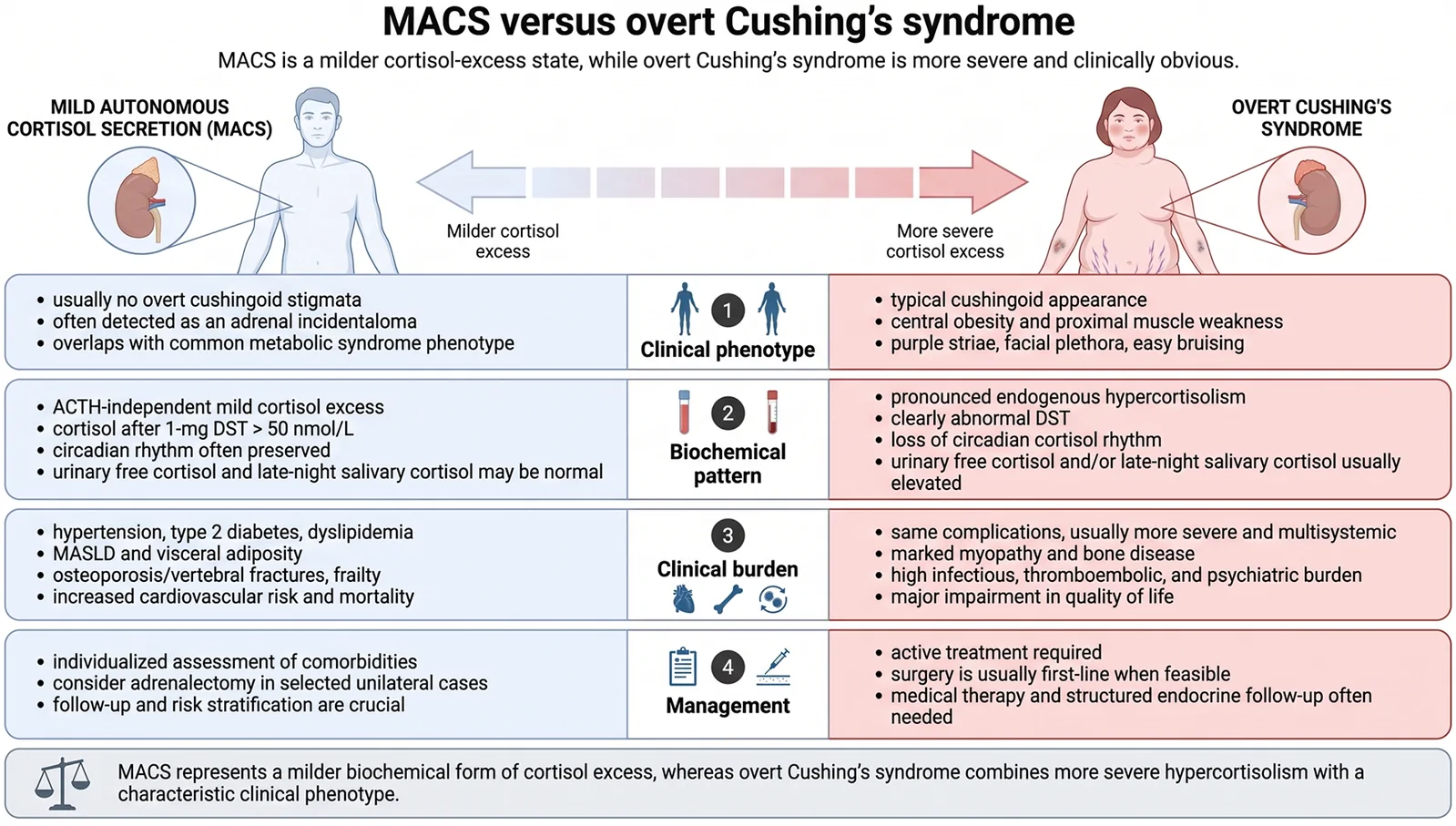
Clinical continuum of cortisol excess: comparison between Cushing’s syndrome and mild autonomous cortisol secretion. ACTH, adrenocorticotropic hormone; CS, Cushing’s syndrome; DST, dexamethasone suppression test; MACS, mild autonomous cortisol secretion; UFC, urinary free cortisol.

Several reviews have summarized the epidemiology and cardiometabolic consequences of MACS. The present review is intended to complement those publications by integrating newer evidence on muscle function and frailty, neurocognitive and sleep outcomes, recent surgical trials, and emerging pharmacological strategies. In addition, we explicitly distinguish evidence obtained directly in MACS from observations extrapolated from overt Cushing’s syndrome or mixed hypercortisolism cohorts, and we provide a practical framework for clinical decision-making. Overall, this review aims to provide clinicians with a comprehensive overview of the multifaceted nature of MACS, while fostering thoughtful consideration of the optimal management and care of affected patients.

## 2 Clinical manifestations and complications of MACS

### 2.1 Cardiovascular complications

An accumulating body of evidence suggests that even low-grade cortisol excess is associated with increased cardiometabolic risk. Compared with patients with NFAT, individuals with MACS are at higher risk of developing new cardiovascular events, including myocardial infarction, stroke, and revascularization procedures (3, 20), as summarized in **Table 1**.

**Table 1:** Summary of key studies on cardiovascular risk in MACS.

Study	Study design	Sample size/population	Key findings
Park et al., 2019 (21)	Systematic Review, 6 studies	n=1,766	New cardiovascular events: 16.7% in MACS vs. 6.7% in NFAT (p=0.04); CV mortality: 22.6% vs. 2.5% (p=0.02).
Elhassan et al., 2019 (7)	Meta-analysis, 32 studies	n=4,121	Hypertension: MACS 64%, NFAT 58%. Cardiovascular: MACS 15.5%, NFAT 6.4%.
Petramala et al., 2020 (22)	Retrospective observational study	n=628 (157 MACS, 471 NFAT)	Higher blood pressure in MACS; myocardial infarction 6.4%, stroke 5.1%, and coronary revascularization 4.5% (all p <0.05); recurrence risk increased during follow-up (RR 2.27-2.41).
Deutschbein et al., 2022 (23)	Retrospective observational study, 28 centers	n=3,656; (1567 MACS, 2089 NFAT)	Hypertension: ACS 75% vs PACS 74% vs NFAT 59% (all p <0.001). Increased mortality with cortisol autonomy (HR 1.77 [95% CI 1.20-2.63] for ACS and HR 1.52 [95% CI 1.19-1.95] for PACS.
Pelsma et al., 2023 (19)	Meta-analysis, 46 studies	n=17156 (6,339 MACS, 10,817 NFAT)	Hypertension more prevalent in MACS than NFAT (RR 1.24 [95% CI 1.16-1.33]); higher incidence of cardiovascular events in cohort studies.

ACS, autonomous cortisol secretion; CI, confidence interval; CV, cardiovascular; HR, hazard ratio; MACS, mild autonomous cortisol secretion; NFAT, non-functioning adrenal tumors; PACS, possible autonomous cortisol secretion; RR, relative risk.

Hypertension is the most common cardiovascular complication associated with MACS, affecting over 60% of patients across multiple studies (24) (**Table 1**). The epidemiologic association is supported directly by MACS cohorts, whereas several proposed mechanisms are derived largely from experimental glucocorticoid biology and overt Cushing’s syndrome. These mechanisms include cortisol-induced sodium retention through mineralocorticoid receptor activation when 11β-hydroxysteroid dehydrogenase type 2 (11β-HSD2) is saturated, increased vascular sensitivity to catecholamines (3), leptin-mediated sympathetic activation (25), stimulation of the renin-angiotensin-aldosterone system, and impairment of nitric oxide-mediated vasodilation (3). These processes may also disrupt circadian blood pressure regulation, contributing to the observed higher prevalence of a non-dipping blood pressure pattern in patients with MACS (22).

MACS has been associated with markers of atherosclerotic cardiovascular disease (**Table 1**). Chronic exposure to mildly elevated cortisol levels promotes endothelial dysfunction and oxidative stress (22, 24). This may be mediated by cortisol-related dysregulation of bioactive lipids regulating endothelial function, including glycerophospholipids, ceramides, and sphingolipids, along with alterations in amino acid pathways, particularly branched-chain amino acids such as valine and isoleucine, which are linked to mitochondrial dysfunction and pro-inflammatory changes (3). Consistent with this, Petramala et al. reported significantly increased carotid intima-media thickness and lower ankle-brachial index in patients with MACS. These findings suggest early atherosclerotic changes that may contribute to the elevated cardiovascular risk observed in MACS (22).

Cardiac structural and functional abnormalities have also been investigated in MACS. In the prospective ITACA cohort, patients with MACS had a higher prevalence of left-ventricular hypertrophy and diastolic dysfunction, and a greater left-ventricular mass index than patients with NFAT (26). These findings provide direct evidence of cardiac remodeling in cortisol hypersecretion, but additional evidence comes from studies of overt Cushing’s syndrome; for example, Maurice et al. (27), demonstrated increased left ventricular mass index, higher ratio of left ventricular mass index to end-diastolic volume and impaired early left ventricular relaxation in patients with active disease compared with controls.

### 2.2 Disorders of glucose and lipid metabolism

Disorders of glucose metabolism are common in MACS affecting approximately 15-40% of patients and manifesting as impaired fasting glucose, prediabetes, or type 2 diabetes mellitus, often accompanied by elevated fasting glucose and glycated hemoglobin (HbA1c) levels (16, 24). The data derived from broader mechanistic glucocorticoid literature show that cortisol increases hepatic glucose production by stimulating gluconeogenic enzymes (including phosphoenolpyruvate carboxykinase and glucose-6-phosphatase), while simultaneously reducing insulin-mediated glucose uptake in peripheral tissues by interfering with insulin signaling Moreover, glucocorticoids inhibit pancreatic β-cell insulin secretion, further contributing to hyperglycemia (28). Key findings from representative studies comparing metabolic abnormalities in MACS and NFAT are summarized in **Table 2**.

**Table 2:** Metabolic abnormalities in MACS versus NFAT across selected studies.

Study	Study design	Sample size/population	Key findings
Kim et al., 2014 (29)	Cohort study	n=237 (19 MACS, 218 NFAT)	Lipids: Total cholesterol 189.9 ± 37.9 vs 170.7 ± 42.9 mg/dL (p =0.048). LDL-C, HDL-C, and triglycerides not significantly different.
Elhassan et al., 2019 (7)	Meta-analysis, 32 studies	n=4121 (784 MACS, 2873 NFAT, 464 NFAT or MACS)	Glucose: T2DM in MACS 28.1% vs NFAT 14.4%
			Lipids: similar baseline prevalence of dyslipidemia (MACS 34.1% vs NFAT 33.8%)
Petramala et al., 2020 (22)	Retrospective cohort study	n=628 (157 MACS, 471 NFAT)	Glucose: T2DM in MACS 19% vs NFAT 7% (p <0.05)
Prete et al., 2022 (30)	Prospective cross-sectional study	n=1305 (65 CS, 591 MACS, 649 NFAT)	Glucose: T2DM in MACS 28.1% vs NFAT 14.4% (aPR 1.62 [95% CI 1.8-2.43])
Deutschbein et al., 2022 (23)	Multicenter cohort study	n=3656 (1567 MACS, 2089 NFAT)	Glucose: T2DM in NFAT 18%, MACS/PACS 23%, ACS 27% (overall p <0.001)
Pelsma et al., 2023 (19)	Meta-analysis	n=17156; (6339 MACS, 10817 NFAT)	Glucose: Increased diabetes risk in MACS (RR = 1.44 [95% CI 1.23-1.70])
			Lipids: Significantly higher risk of dyslipidemia in MACS compared with NFAT (RR 1.29).
Yu et al., 2024 (16)	Cross-sectional study	n=262 (59 CS, 100 MACS, 103 NFAT)	Glucose: higher fasting glucose in MACS 8.20 vs NFAT 5.58 mmol/L (p <0.001) and HbA1c in MACS 6.85% vs NFAT 6.20% (p =0.005)
			Lipids: Triglycerides in MACS 2.28 vs NFAT 1.63 mmol/L (p < 0.001), and LDL-C: MACS 3.12 vs NFAT 2.34 mmol/L (p < 0.001).
			Liver: higher NAFLD prevalence in MACS 57.0% vs NFAT 26.2% (p <0.001). Liver-to-spleen ratio: MACS 0.953 vs NFAT 1.198 (p <0.001).
Erdogan et al., 2025 (31)	Retrospective cohort study	n=461 (77 MACS, 384 NFAT)	Glucose: T2DM in MACS 42.9% vs NFAT 28.9% (p =0.016)
Candemir et al., 2025 (32)	Cohort study	n=100 (40 MACS, 60 NFAT)	Lipids: triglycerides in MACS 1.79 ± 0.47 vs NFAT 1.49 ± 0.52 mmol/L (p =0.004); LDL-C and HDL-C did not differ significantly.
			Liver: CT-defined NAFLD more prevalent in MACS than NFAT

ACS, autonomous cortisol secretion; aPR, adjusted prevalence ratio; CI, confidence interval; CS, Cushing’s syndrome; CT, computed tomography; HbA1c, glycated hemoglobin; HDL-C, high-density lipoprotein cholesterol; LDL-C, low-density lipoprotein cholesterol; MACS, mild autonomous cortisol secretion; NAFLD, nonalcoholic fatty liver disease; NFAT, non-functioning adrenal tumors; PACS, possible autonomous cortisol secretion; RR, relative risk; T2DM, type 2 diabetes mellitus.

Dyslipidemia is highly prevalent in MACS, with reported rates ranging from 26% to 67% (24). Meta-analytic data suggest a significantly higher risk of dyslipidemia in MACS than in NFAT. However, individual lipid fractions are heterogeneous across studies; several studies reported higher triglycerides and/or low-density lipoprotein cholesterol (LDL-C) in MACS, whereas others found smaller or nonsignificant differences. The detailed lipid findings are presented in **Table 2**.

MACS has also been associated with metabolic dysfunction-associated steatotic liver disease (MASLD), likely due to cortisol-induced activation of glucocorticoid receptors in adipose tissue, which promotes lipolysis and increases free fatty acid flux to the liver, driving hepatic fat accumulation and insulin resistance (33). This association is supported by imaging-based studies demonstrating increased hepatic fat accumulation and a high prevalence of MASLD in MACS, as shown in **Table 2**.

### 2.3 Musculoskeletal system involvement

Glucocorticoid excess can disrupt skeletal homeostasis (34, 35), but the strength of evidence differs by level of cortisol excess. Experimental and overt-Cushing’s syndrome studies show inhibition of osteoblast differentiation and activity, apoptosis of osteoblasts and osteocytes, and prolonged osteoclast survival (34–36); these mechanisms are biologically plausible in MACS but are not all demonstrated directly at mild cortisol excess. By contrast, direct MACS data show altered bone-turnover markers: in a cross-sectional study of 213 patients with adrenal adenomas, circulating sclerostin concentrations were significantly lower in MACS than in NFAT (35). Conversely, the postoperative increase in sclerostin and fibroblast growth factor 23 reported in 12 patients with overt hypercortisolism (37) support the concept that chronic exposure to glucocorticoids directly impairs osteocyte function. Baseline calcium and parathyroid hormone appear normal in MACS (n=735) (38), whereas changes after adrenalectomy have been reported in small MACS cohorts (39).

The cumulative impact of these mechanisms may induce measurable structural alterations of bone. In a large cohort (n=444), MACS was linked to a significantly higher prevalence of vertebral fractures in women, regardless of menopausal status (odds ratio [OR]=5.36), and men (OR = 5.23), despite the exclusion of patients with hypogonadism (40). A meta-analysis demonstrated lower bone mineral density values at the lumbar spine (3 studies, n=917) and femoral neck (2 studies, n=870) in MACS compared with NFAT, although these differences became significant only after sensitivity analysis excluding an outlier study (17). The lumbar spine is particularly vulnerable to glucocorticoids due to its high trabecular content and metabolic activity (41). However, densitometry may underestimate bone damage; therefore, attention has shifted toward bone quality measures such as trabecular bone score (TBS) (42). Indeed, significant deterioration in TBS was observed in small studies in MACS patients (42, 43). These structural and microarchitectural changes may translate into skeletal outcomes. In a large study by Pal et al., vertebral fractures affected 45% of patients with MACS, with significantly higher odds compared with the NFAT group (OR = 2.10) (17). Given that patients with AIs are often older, fracture consequences, including chronic pain, reduced mobility, loss of independence, and increased morbidity, may substantially impair quality of life (44). In a prospective randomized study (n=53), adrenalectomy significantly reduced the incidence of vertebral fractures compared with conservative management (4.8% vs 25%) (39).

Muscle function may represent another clinically relevant target of mild cortisol excess. A recent cross-sectional study (n=138) evaluating patients with AIs demonstrated reduced muscle mass, function, and quality, particularly in women, with more pronounced impairment among patients with MACS (45). Similar observations were reported in a study (n=62) comparing MACS, non-functioning adrenal adenomas, and healthy controls, in which MACS patients showed lower biceps and quadriceps strength on the Medical Research Council scale despite comparable fat-free mass, muscle mass, and fat mass, suggesting that muscle dysfunction may occur even in the absence of overt sarcopenia (46). The clinical relevance of this finding is supported by interventional data (n=40) showing that adrenalectomy was associated with significant improvement in handgrip strength, while conservative management was followed by a decline in grip strength over 6 months (47).

### 2.4 Thromboembolic risk

Overt Cushing’s syndrome is associated with a well-established prothrombotic state and increased venous thromboembolism (VTE) risk which is reflected by the current recommendation paper by Isand et al. Glucocorticoids in CS increase circulating levels of procoagulant factors (e.g., fibrinogen, factor VIII, von Willebrand factor) and alter fibrinolytic activity (48, 49). Enhanced expression of plasminogen activator inhibitor-1 contributes to impaired clot resolution (50, 51). Whether the same abnormalities translate into a clinically meaningful increase in VTE specifically in MACS remains uncertain, although some studies suggest that attenuated procoagulant changes may potentially contribute to the increased cardiovascular risk observed in MACS (30, 52). Beyond these potential direct effects on coagulation pathways, the burden of cardiovascular and metabolic comorbidities observed in MACS, including hypertension, impaired glucose tolerance or type 2 diabetes mellitus, central obesity, insulin resistance and dyslipidemia, may further contribute to increased thromboembolic risk (3, 53–55).

In overt CS, the incidence of VTE is markedly elevated compared with the age-matched general population (56). Despite the lack of robust prospective data on VTE in MACS, results from a large retrospective cohort study by Morelli et al. showed a significantly increased risk of cardiovascular events, with pulmonary embolism included among the events assessed, regardless of age, smoking, and cardiometabolic comorbidities (52).

Thromboembolic risk stratification in MACS should incorporate traditional cardiovascular, biochemical, and perioperative factors (48). Consideration of thromboprophylaxis in high-risk settings may be warranted, although standardized recommendations are lacking (50, 56). Adrenalectomy in cortisol-secreting adrenal adenomas may partially normalize coagulation profiles in overt CS (50, 51); however, data addressing changes in thromboembolic incidence in MACS remain scarce, underscoring the need for longitudinal studies (1).

### 2.5 Frailty

Frailty, like delirium or falls, is a “geriatric syndrome” common in older adults and caused by multiple system impairments leading to functional decline (57). There is no universal definition of frailty, although the Fried frailty phenotype (presence of at least three of the five criteria: unintentional weight loss, self-reported exhaustion, weakness (assessed by grip strength), slow walking speed, and low physical activity) and frailty indexes are widely used (58). Frailty, mainly affecting people over 65, increases risk of falls, disability, hospitalization, and mortality, making early identification essential (59).

Frailty reflects accelerated molecular aging driven by inflammation and hormonal imbalance, including a decline in anabolic hormones and relative cortisol excess, leading to reduced resilience (60). In observational MACS cohorts, frailty is more frequent than in NFAT (61), and MACS is associated with lower muscle mass (62), increased visceral adiposity (62, 63) and lower muscle strength (64) – factors which may contribute to functional limitations. Presumably, a delayed diagnosis of MACS related to the absence of overt hypercortisolism may prolong exposure time and accelerate premature aging (65). These data do not establish that mild cortisol excess directly causes frailty; age, multimorbidity, and other metabolic factors may contribute substantially.

A dedicated frailty index consisting of 47 variables, yielding a cumulative value from 0 to 1, with a cut-off of 0.25 defining frailty, was created to assess patients with MACS (61) and retrospectively validated in 443 patients (275 NFAT, 168 MACS). Frailty was significantly more common in MACS (24% vs 18%), particularly in those ≥65 years. MACS patients also more frequently reported limb weakness, falls, and sleep disturbances, contributing to increased frailty (61). This index was also validated prospectively, on 520 patients with NFAT (n=163), MACS (n=212), CS (n=64), primary aldosteronism (n=81), and referent subjects (n=89) (65). Frailty was significantly more frequent in patients with hormone-secreting adenomas, with the highest frequency in CS (59%), and MACS (42%) when compared to controls (4%) and the NFAT group (22%). MACS patients more commonly reported musculoskeletal pain/stiffness, falls, gait assistance and inability to climb two flights of stairs. The index score correlated with functional performance measures, including handgrip strength and the 6-minute walk test. A dedicated study comparing MACS (n=81) and CS (n=83) showed reduced handgrip strength in MACS versus healthy controls, but comparable strength to CS in handgrip and sit-to-stand tests (64). An emerging area is the identification of possible laboratory biomarkers of frailty, particularly markers of chronic inflammation and coagulation, such as interleukin-6 (“geriatric factor”), C-reactive protein, tumor necrosis factor alpha, D-dimer, fibrinogen, lymphocyte count, soluble tumor necrosis factor receptors, and intercellular adhesion molecule-1 (66).

The effect of adrenalectomy on frailty among patients with MACS remains uncertain, because available interventional evidence is limited. A single-center prospective study (n=41) demonstrated a significant reduction in frailty index post-adrenalectomy, without improvement in muscle performance (gait speed, 6-minute walk, handgrip) (67). Conversely, a multicenter prospective study (n=253) showed significantly greater frailty improvement in surgically treated patients (n=108) versus conservative management, adjusted for age and sex (68). Although the effect of surgery remains uncertain, systematic frailty screening may guide management through targeted rehabilitative interventions (69).

### 2.6 Circadian rhythms, sleep disturbances, psychiatric and cognitive function

The hypothalamic-pituitary-adrenal (HPA) axis, through corticotropin-releasing hormone, ACTH, and cortisol, regulates circadian rhythms and the sleep-wake cycle (70). The hypothalamic suprachiasmatic nucleus drives the 24-hour rhythm in cortisol secretion, with a morning peak and gradual daytime decline (71). Cortisol synchronizes peripheral clocks through glucocorticoid receptor–mediated regulation of core clock genes, e.g., basic helix-loop-helix ARNT like 1 (*Bmal1*), cryptochrome circadian regulator 1 (*Cry1*), period circadian regulator 1 (*Per1)*, period circadian regulator 2 (*Per2*) (72). Longstanding hypercortisolism in CS changes clock gene expression and epigenetic regulation, including histone modifications (73).

Higher cortisol exposure has been associated with sleep disturbances in observational studies: elevated late-night salivary cortisol correlates with shorter sleep duration and reduced efficiency (74), higher daytime cortisol is associated with a higher sleep-fragmentation score and longer wake after sleep onset (75), whereas elevated urinary cortisol is associated with lower sleep efficiency and an altered sleep-phase pattern (76). In overt CS and MACS, sleep and psychiatric abnormalities have also been reported (77–79), but the casuality is difficult to establish because sleep disruption itself can alter HPA-axis activity and because age, obesity, mood disorders, and sleep apnea may confound these associations.

A cross-sectional study comparing CS (n=154), MACS (n=194), and controls (n=89) using the Pittsburgh Sleep Quality Index (PSQI) found that MACS patients had shorter sleep duration, longer latency, lower efficiency, and worse daytime function than controls (80). These abnormalities were associated with increased use of sleep medications and were more common in younger women. Interestingly, the differences in PSQI and its subcomponents were not statistically significant between MACS and CS patients in age-, sex-, and body mass index-adjusted analysis. In MACS, the degree of clinical severity of hypercortisolism assessed by the clinical severity score was associated with impaired sleep, which was not observed in CS. Obstructive sleep apnea was significantly more prevalent in MACS (45% vs. 19%) but was not associated with sleep quality.

Psychiatric disturbances appear to be more common in MACS, as reflected by higher scores in the Beck Depression Inventory-II scale than in healthy controls (81), and by a higher incidence of mood disturbances, anxiety, irritability and reduced interest compared with NFAT patients (65).

Cortisol participates in memory and cognitive processes (82, 83), and elevated cortisol has been associated with Alzheimer’s disease, reduced hippocampal volume, and impaired memory (84). Only a few studies have assessed cognitive function in MACS and their findings remain inconsistent (**Table 3**). Morelli et al. reported better performance in selected cognitive domains, including verbal fluency, symbol coding and executive functions. The authors hypothesized that these functions, which depend on prefrontal cortical activation, might be facilitated by relatively mild glucocorticoid exposure such as that observed in MACS (85). In contrast another study found no significant differences in neuropsychological test performance despite structural thalamic alterations (86), while other reported poorer cognitive outcomes, including impaired working memory or composite cognitive scores in MACS (87, 88). These discrepancies may reflect small and heterogeneous cohorts, differences in age and comorbidity burden, variable definitions and biochemical severity of MACS, use of different cognitive batteries and domain-specific endpoints, and unequal adjustment for confounders. Longitudinal data after adrenalectomy remain limited, so current studies support an association between MACS and selected cognitive alterations rather than a uniform causal neurocognitive phenotype.

**Table 3:** Cognitive function in MACS: summary of selected studies.

Study	Study design	Sample size/population	Key findings
Morelli et al., 2021 (85)	Prospective, multicenter study	n=62 AI (43 MACS, 19 NFAT); cognitive subgroup n=26; post-adrenalectomy n=8	MACS: better verbal fluency (p = 0.012), symbol coding (p = 0.013) and executive functions (0.009). No significant changes after adrenalectomy.
Sulu et al., 2023 (86)	Cross-sectional study	n=92 (23 MACS, 21 active CS, 27 CS in remission, 21 controls)	Smaller thalamic volumes in MACS and active CS than controls. Smaller left medial and right lateral thalamic volumes in CS in remission. No significant differences between MACS, CS and controls in neuropsychological tests
Liu et al., 2023 (87)	Prospective study	n=63 AI (27 MACS, 36 NFAT)	Higher frequency of subjective memory complaints (40.7% vs 13.9%, p<0.05), higher frequency of mild cognitive impairment (22.2% vs 2.8%, p<0.05), and worse working memory in patients with MACS. Post 1 mg DST cortisol as a risk factor for impairment of working memory in multivariate linear regression analysis (p < 0.05)
Saini et al., 2024 (88)	Cross-sectional and longitudinal study using	n=285 (84 MACS and 201 referent subjects; post-adrenalectomy n=37)	Lower cognition in MACS due to lower total composite and fluid composite cognitive scores (p < 0.001) but not crystallized composite score (p = 0.130). A small increase in some cognitive functions post-adrenalectomy.

AI, adrenal incidentaloma; BDI, Beck Depression Inventory; CS, Cushing’s syndrome; DST, dexamethasone suppression test; MACS, mild autonomous cortisol secretion; MRI, magnetic resonance imaging; NFAT, non-functioning adrenal tumors; NIH, National Institutes of Health; SF-36, 36-Item Short Form Health Survey.

### 2.7 Hypogonadism

While secondary hypogonadism is well documented in overt CS, evidence in MACS remains limited to small observational cohorts. In a study by Nowak et al., including 17 men with MACS and 30 men with overt CS, hypogonadism was present in 59% of MACS patients and 93% of CS patients. Total testosterone level was lower in men with MACS than in matched controls (9.4 nmol/L vs 12.4 nmol/L), but this difference was not statistically significant (89). A subsequent retrospective analysis of 25 men with MACS and 38 men with NFAT reported lower total testosterone (90). These results suggest that mild cortisol excess may be associated with the suppression of the hypothalamic-pituitary-gonadal axis and support consideration of gonadal axis assessment in men with MACS, particularly in the presence of suggestive symptoms (90).

### 2.8 Gut microbiota

Dysbiosis, an imbalance in the gut microbial composition and functionality, has been increasingly implicated in the pathogenesis of cardiometabolic and endocrine disorders, including obesity, insulin resistance, and atherosclerosis (91, 92). Moreover, specific genera of gut microorganisms can modulate adrenal function by influencing hormone synthesis, although the exact mechanisms of these interactions remain unknown (93). No study has directly characterized gut microbial composition in patients with MACS. Current hypotheses are derived mainly from overt Cushing’s syndrome, in which alterations in microbial composition have been reported. CS patients show reduced *Bacteroidetes*, increased *Firmicutes*, *Actinobacteria*, and *Proteobacteria* abundance, presence of cortisol-metabolizing bacteria *Ruminococcus gnavus* (94) and an overall shift toward proinflammatory microbial signatures (95). This microbial profile is often linked with obesity (96), endotoxemia and insulin resistance (97). The dysbiosis in CS may persist after treatment and is considered a potential factor contributing to the elevated cardiovascular risk observed in CS patients even after biochemical remission (98, 99). Whether similar changes occur in MACS remains unknown.

One study evaluated selected microbiome-derived metabolites in MACS (n = 213) by measuring plasma concentrations of trimethylamine N-oxide (TMAO) and betaine, molecules contributing to inflammation and cardiovascular risk. Patients had higher betaine concentrations in adjusted analysis (OR 1.22, 95% CI 1.04-1.44), while plasma TMAO concentration was not significantly associated with MACS (OR 1.02, 95% CI 0.87-1.20). It is suggested that higher betaine concentrations may reduce inflammation, suggesting that the gut microbiome, acting through its metabolites, may attenuate the metabolic effects of subclinical hypercortisolism (100).

### 2.9 MACS and other adrenal disorders

Mild cortisol hypersecretion may coexist with other adrenal endocrine disorders rather than occurring exclusively in otherwise non-functioning adrenal adenomas. In primary aldosteronism (PA), a 2025 retrospective cohort study with systematic review/meta-analysis reported a prevalence of concomitant MACS of 21.9%. Patients with PA and MACS were older and had larger adrenal tumors, higher aldosterone concentrations, more suppressed renin, lower estimated glomerular filtration rate, and a higher prevalences comorbidities than PA patients without MACS (101). These observations support active consideration of cortisol co-secretion in PA, while the incremental causal contribution of mild cortisol excess to those comorbidities remains uncertain.

Abnormal cortisol suppression has also been reported in pheochromocytoma and paraganglioma (PPGL). In a retrospective two-center cohort of 106 patients with PPGL, 24.5% had non-suppressed cortisol after the 1 mg DST. These patients were characterized by predominantly female sex, older age, larger tumors, higher metanephrine concentrations, comorbidity and increased perioperative risk (102). Another multicenter Korean study of pheochromocytoma similarly reported non-suppressed post-DST cortisol in approximately 37% of patients (103). Importantly, non-suppression in PPGL should not automatically be equated with classic adrenal MACS, because catecholamine-related HPA-axis activation, stress physiology, and tumor context may influence DST results. The pathophysiology and clinical implications of cortisol dysregulation in PPGL therefore require prospective clarification.

## 3 Diagnostics

All patients with newly diagnosed adrenal incidentalomas should be screened using the 1 mg overnight DST (1). A post-DST serum cortisol concentration >1.8 μg/dL (>50 nmol/L), in the absence of signs of overt Cushing’s syndrome, is the current guideline criterion for MACS, while values ≤1.8 μg/dL generally exclude autonomous cortisol secretion (3). Although the guidelines do not recommend routine additional testing, several aspects should be considered. DST interpretation requires exclusion of common causes of false-positive results, including poor adherence to dexamethasone, cytochrome P450 3A4‑inducing drugs (e.g., carbamazepine, rifampicin), impaired gastrointestinal absorption, elevated cortisol-binding globulin, and chronic renal failure (104). Measuring serum dexamethasone can confirm test validity; in one cohort (n=58), inadequate dexamethasone levels explained 22% of positive DST results, increasing misclassification risk (105). Although combined cortisol and dexamethasone assessment may improve accuracy, its routine use remains limited (104). Despite guideline recommendations against repeat testing after normal results unless new signs emerge (1), multicenter data (n=2525) showed DST changes in 22.5% of patients with benign AIs, including 9% progressing from NFAT to MACS (106). A recent multicenter Korean study further demonstrated that post-DST cortisol levels may increase over time, with a progressive rise in overnight DST ratios, suggesting dynamic changes in cortisol secretion and a potential need for longitudinal reassessment in selected patients (107). When tandem mass spectrometry is used, no single universal liquid chromatography-tandem mass spectrometry (LC-MS/MS)-specific cortisol or dexamethasone cutoff has been validated across laboratories. Method-specific thresholds should be interpreted against the local assay and reference population. Exogenous glucocorticoid use should be carefully evaluated, as commonly overlooked inhaled, nasal, and topical preparations may interfere with cortisol testing and yield false-negative results.

Once hypercortisolism is confirmed, ACTH independence should be determined. In overt CS, a morning plasma ACTH <10 pg/mL suggests an adrenal cause, >20 pg/mL indicates ACTH-dependent disease, and 10–20 pg/mL is indeterminate. In MACS, clear ACTH cut-offs are lacking; however, in a study of 371 patients, basal ACTH <10 pg/mL supported a diagnosis of MACS with 75.3% specificity and 77.9% positive predictive value (108). Interpretation is limited, as ACTH may be suppressed by factors other than hypercortisolemia (109). Therefore, a post-DST ACTH <0.6 pmol/L and a dexamethasone suppression ratio <18% have been proposed as more reliable markers of ACTH independence, though further validation is needed (110).

Dehydroepiandrosterone sulfate (DHEA-S) may serve as a marker of chronic HPA-axis suppression when low. Owing to minimal diurnal variation, it can support the evaluation of equivocal cases and the estimation of cortisol excess, although its use is limited by a lack of standardized age-adjusted reference ranges (1). In a study of 468 patients (ACTH available in 371), Carafone et al. showed that DHEA-S and ACTH have comparable diagnostic accuracy for MACS, with DHEA-S demonstrating higher specificity and positive predictive value; however, interpretation is limited by the retrospective design and potential misclassification due to false-positive DST results. The study also did not confirm superiority of the sex-adjusted DHEA-S ratio. A meta-analysis of seven studies identified a basal DHEA-S <60–70 μg/dL as providing the best sensitivity and specificity for MACS (111). The highest diagnostic accuracy appears to be achieved by combining DHEA-S with ACTH measurement (108).

Given the limitations of DST, other tests for hypercortisolism have been evaluated. Late-night salivary cortisol (LNSC) and urinary free cortisol (UFC), although standard in overt CS, lack sensitivity for MACS. Because circadian rhythm is often preserved in MACS, LNSC may remain normal, with reported sensitivity of only 23–40%, limiting its ability to distinguish non-functioning from hormone-secreting tumors (112–114). The diagnostic performance of UFC is even poorer, rendering it not useful (112).

Beyond the guideline-based DST and supportive ACTH/DHEA-S assessment, several biomarkers are being investigated to capture cumulative or tissue-level glucocorticoid exposure. Hair cortisol and cortisone reflect cumulative exposure over weeks to months. While blood and salivary tests reflect only single time points, methods to assess long-term tissue exposure to glucocorticoids are being developed. Hair analysis has emerged as a promising candidate, as cortisol is passively incorporated into the hair shaft from the circulation during its growth (115, 116). In overt CS, hair cortisol and cortisone levels are both robust diagnostic indicators (116, 117). In MACS, hair cortisone concentration emerges as the more discriminative biomarker, achieving a high specificity of 94% (117). Nonetheless, its modest sensitivity of 68% represents a critical limitation as a reliable rule-out test in clinical practice, where a significant proportion of true cases may be missed.

Similarly, salivary cortisone presents an analytical advantage: as the inactive 11-keto metabolite of cortisol, its measurement is not confounded by topical hydrocortisone contamination. In transit to the oral cavity, the majority of circulating cortisol is converted to cortisone by 11β-HSD2 in the salivary glands (118). Consequently, cortisone predominates in saliva, with a cortisol-to-cortisone ratio reversed from approximately 5:1 in serum to 1:5 (119). Although its diagnostic performance in adrenal incidentaloma cohorts is promising, with a sensitivity of 90% and specificity of 91% for identifying MACS (113), the required quantification by LC-MS/MS limits widespread clinical adoption and standardization. Consequently, late-night salivary cortisone remains an investigational biomarker, rather than an established component of routine diagnostic practice.

Limited availability of advanced diagnostics underscores the need for accessible biomarkers. Routine hematological parameters have emerged as potential surrogates of chronic glucocorticoid exposure. Cortisol-induced immunosuppression leads to characteristic changes in white blood cells, reflected by neutrophilia, lymphopenia, and reduced eosinophil and basophil counts, correlating with cortisol levels in a dose-dependent manner. These alterations vary by etiology; for instance, ectopic CS shows more pronounced changes than ACTH-independent disease, potentially supporting differentiation between etiological subtypes (120). A recent study proposed a diagnostic model to differentiate MACS from NFAT using routine blood count parameters (121). While neutrophil-to-lymphocyte ratio alone showed limited accuracy (area under the curve [AUC] 0.629), a classification and regression tree model incorporating 14 leukocyte variables markedly improved performance (AUC 0.872; sensitivity 95%, specificity 75.7%). Rather than serving as a definitive diagnostic criterion, this approach may be primarily useful for initial risk stratification and exclusion of MACS. Accordingly, current research (summarized in **Table 4**) is increasingly focused on identifying and refining novel diagnostic biomarkers. Importantly, several of the most recent biomarker findings summarized in **Table 4** have been reported only in conference abstracts, with limited methodological details and without independent validation. These findings should therefore be considered preliminary and interpreted with caution. Further prospective studies with standardized methodologies and external validation are required before these biomarkers can be incorporated into routine clinical practice.

**Table 4:** New biomarker approaches potentially relevant for future risk stratification and treatment selection in MACS.

Study	Study design/evidence source	Sample size/population	Evidence level	Validation status	Key findings
Prete et al., 2026 (122)	Multicenter urinary steroid and serum metabolomics	Urine: n=1305 (591 MACS, 649 NFAT, 65 CS), Serum: n=291 (140 MACS, 104 NFAT, 47 CS).	Emerging multicenter biomarker research	Promising association data; clinical cutoffs not validated	Higher urinary GC metabolites and 11-oxygenated androgens correlated with hypertension and type 2 diabetes. Gradual shift in serum metabolomics toward a more lipotoxic profile from NFAT to MACS and CS.
Galante et al., 2025 (123)	Circadian metabolomics from dried blood spots; conference abstract	n=39 (12 MACS, 9 CS, 8 AI, 10 controls)	Early exploratory	No independent validation	Cortisol excess disrupted circadian metabolic rhythms. Phosphatidylcholines differentiated MACS and CS from healthy controls, most prominently in the afternoon (p<0.05).
Suchy et al., 2025 (124)	Retrospective analysis of CBC-derived inflammatory scores; conference abstract	n=603 (121 MACS, 184 CS, matched NFAT controls)	Early exploratory	No independent validation	Systemic inflammation scores (e.g., NPR, PLR, SII) correlated significantly (p<0.05) with liver fibrosis risk (FIB-4).
Crastin et al., 2026 (125)	*In vitro* macrophage immunometabolic profiling using patient serum; conference abstract	n=28 (12 MACS, 6 CS, 10 NFAT)	Mechanistic/translational	No clinical validation	MACS serum suppressed M1-like macrophage activation, impaired phagocytosis (p=0.014), and showed a trend toward increased mitochondrial viability (p=0.059).
Glinicki et al., 2025 (126)	Urinary steroid and blood immunochemical profiling, conference abstract	n=61 (26 MACS, 7 CS, 9 PA, 19 NFAT)	Early exploratory	No independent validation	- Androsterone (p=0.008) and androstane-3-ol (p=0.002) were higher in NFAT, - tetrahydro-11-deoxycortisol (p=0.009) was higher in MACS Adding chromogranin A improved diagnostic performance mainly for overt CS.

AI, adrenal incidentaloma; CBC, complete blood count; CS, Cushing’s syndrome; FIB-4, fibrosis-4 index; GC, glucocorticoid; MACS, mild autonomous cortisol secretion; NFAT, non-functioning adrenal tumors; NPR, neutrophil-to-platelet ratio; PA, primary aldosteronism; PLR, platelet-to-lymphocyte ratio; SII, systemic immune-inflammation index.

## 4 Treatment

### 4.1 Surgery

The main challenge after MACS diagnosis is determining whether cortisol excess warrants treatment or observation. The current guidelines suggest surgery may be considered in patients with MACS and relevant comorbidities when the adrenal mass is unilateral, taking into account age, general health status, comorbidities, persistence of cortisol excess, and patient preference. If chosen, a minimally invasive adrenalectomy performed by an experienced surgeon is recommended, with perioperative glucocorticoid coverage and endocrine follow‑up to ensure HPA axis recovery.

In bilateral MACS, treatment should be individualized with a focus on managing cortisol‑related comorbidities (1). A recent large international retrospective cohort study including 629 patients with bilateral masses (83% with MACS and 17% with CS) demonstrated that surgery was used in only 27% of MACS patients and either conservative or pharmacological management was favored, leading to heterogeneous biochemical outcomes and no substantial improvement in comorbidities (127).

Historically, the management of MACS relied almost entirely on observational evidence, expert consensus, and non-randomized studies (summarized in **Table 5**). A meta-analysis by Bancos et al., incorporating more than 500 MACS patients from 26 studies, showed that adrenalectomy significantly improved blood pressure outcomes compared with conservative therapy (relative risk [RR] 11, 95% CI 4.3–27.8) (131). A more recent analysis by Pelsma et al., including 30 cross-sectional and 16 cohort studies with 17,156 patients, further confirmed these findings in the context of blood pressure control (RR 8.8). Selected key studies are presented in **Table 5**. Recent years have brought several randomized controlled studies (**Table 6**), and although still limited in scope and number, they have substantially strengthened the evidence on the surgical management of MACS.

**Table 5:** Selected non-randomized comparative studies evaluating adrenalectomy versus conservative management in patients with MACS.

Study	Study design	Sample size/population	Key findings
Salcuni et al., 2016 (128)	Prospective, single-center study	n=55, unilateral AIs and MACS (32 adrenalectomy, 23 conservative)	In adrenalectomy group: -lower incidence of new vertebral fractures (9.4% vs. 52.2%, P<0.0001) and an independent reduction in fracture risk compared with conservative management.
Araujo-Castro et al., 2022 (129)	Retrospective, single-center study	MACS: 42 surgery, 217 control; NFAT: 16 surgery, 470 control	In adrenalectomy group: -greater decrease in glucose levels (-16.6 ± 45.07 vs. -1.0 ± 26.92 mg/dL, P = 0.035) and in triglycerides (-20.21 ± 55.97 vs. 1.3 ± 59.23 mg/dL, P = 0.029) Improvement in systolic blood pressure also seen in surgically treated NFAT patients (-11.1 ± 15.94 vs 1.0 ± 17.54 mmHg, P = 0.009) vs conservatively treated patients.
De Alcubierre et al.,2026 (26)	Prospective, single-center study	n=60 total (35 MACS: 22 surveillance, 13 adrenalectomy; 25 NFAT)	MACS patients had a greater LV mass index (LVMi) that decreased after adrenalectomy, but rebounded after 5 years.
Morelli et al., 2025 (39)	Retrospective comparative intervention study	n=53 MACS (31 surgery, 22 conservative)	In adrenalectomy group: -fewer vertebral fractures than conservative group (9.7% vs 50%, P < 0.005).
Kumar et al., 2026 (130)	Retrospective comparative cohort study	53 MACS after adrenalectomy; 12 NFAT controls	In MACS adrenalectomy group: – More frequent weight improvement than controls (51% vs 11%, P = .03). – No significant differences in postoperative blood pressure improvement (58% vs 73%, P = .38). – No significant improvement in HbA1c (6.1%→6.2%, P = .46). – No significant improvement in lipid profile (LDL 92.9→85.3 mg/dL, P = .08).

AIs, adrenal incidentalomas; HbA1c, glycated hemoglobin; LDL, low-density lipoprotein; LV, left ventricular; LVMi, left ventricular mass index; MACS, mild autonomous cortisol secretion; NFAT, non-functioning adrenal tumors.

**Table 6:** Randomized clinical trials comparing surgical approach versus observation in patients with MACS.

Study	Study design	Sample size/population	Key findings
Toniato et al.,2009 (132)	Randomized, single-center study	n=45 MACS (23 surgery, 22 conservative)	In adrenalectomy group improvement in: -glycemic control (62.5% (5/8) vs worsening in 2/6 control patients, p=0.619) -hypertension (67% (12/18) vs worsening in 12/15 control patients, p=0.046) - obesity (50% (3/6), no p-value reported)
Ueland et al.,2025 (133)	Randomized clinical trial; 5 centers in Sweden	n=43 MACS (21 adrenalectomy, 22 conservative)	In adrenalectomy group: -improved office blood pressure and daily defined doses of antihypertensives in 75% vs 44% (P = 0.01). -decreased glucose level during OGTT in patients with T2DM (6.2 vs 7.3 mmol/L, P = 0.04) -no statistically significant differences in QoL between the two groups.
Tabarin et al. CHIRACIC Study, 2025 (134)	Open-label, parallel randomized controlled trial; 17 European centers; follow-up to 13 months	n=49 (23 adrenalectomy, 26 conservative)	In adrenalectomy group significant reduction in: -systolic blood pressure during 24 h ABPM - antihypertensive treatment with normal HBPM 46% vs 15%, p=0·0038 - the number of individuals still needing antihypertensive treatment – 43% vs. 96%, p<0·0001
Morelli et al.,2022 (128)	Randomized multicenter intervention study	n=62 AI with possible autonomous cortisol secretion	In adrenalectomy group: -improved blood pressure control (68% vs 13%, p = 0.001) -improved glycometabolic control (28% vs. 3.3%, p = 0.02)
COAR study, 2024 (135)	Prospective multicenter randomized study; median follow-up 48 months	n=132 MACS (66 adrenalectomy, 66 control)	In adrenalectomy group; improved: -weight control (32,6% vs 6,5%, P = 0.002) -glucose control (45,7% vs 15,2%, P = 0.002) -blood pressure control (45,7% vs 23,9%, P = 0.029)

ABPM, ambulatory blood pressure monitoring; AIs, adrenal incidentalomas; COAR, Co-work of Adrenal Research; HBPM, home blood pressure monitoring; MACS, mild autonomous cortisol secretion; OGTT, oral glucose tolerance test; QoL, quality of life; T2DM, type 2 diabetes mellitus.

Interventional studies suggest that the greatest benefits of surgery are observed in patients with clinically significant cortisol-related comorbidities, particularly those of recent onset (39, 134–136). However, in the large multicenter cohort of 3656 patients with incidentalomas, women aged <65 years with MACS (specifically with post-DST cortisol >138 nmol/L) had the highest mortality risk compared with patients with NFAT (adjusted HR 4.37), whereas men aged ≥65 years showed no increased mortality risk (HR 1.09) (23). The authors suggested that women under the age of 65 may benefit most from normalizing cortisol secretion, although this requires confirmation in interventional trials (23).

Postoperative adrenal insufficiency due to suppression of the contralateral adrenal gland by chronic ACTH suppression is a relatively common and clinically important issue after adrenalectomy in patients with MACS (1). However, its true frequency, diagnostic approach, and expected recovery time are still not clearly defined. Differences between studies are largely due to changing definitions of MACS and the use of varying biochemical criteria to diagnose postoperative adrenal insufficiency after surgery. Typically, a cutoff of 10 μg/dL is used for cortisol values to distinguish patients with suspected postoperative adrenal insufficiency from those with preserved adrenal function.

A large study on 281 patients with MACS by Hamidi et al. found that over half of the patients (54.5%) developed postoperative adrenal insufficiency after unilateral adrenalectomy (137). The risk was higher in younger individuals, women, patients with larger tumors, higher post-DST cortisol levels, and lower ACTH levels before surgery. Importantly, the severity of hormonal disturbances before surgery helped predict both the likelihood of adrenal insufficiency and the time needed for recovery. On average, recovery took about 4 months but ranged from around 3 months in milder cases to more than one year in more severe ones.

In contrast, in a study including 139 MACS patients, Ibrahimli et al. reported a much lower proportion of patients requiring steroid replacement (23%) (138). In their study, patients were assessed using a structured postoperative protocol including cortisol measurement on day 1, ACTH stimulation testing, and clinical evaluation. The main predictors of needing glucocorticoid therapy were very low ACTH (<7 pg/mL) and higher cortisol levels after DST (>4.2 μg/dL) before surgery. Notably, all patients were eventually able to stop steroid treatment, typically within about 2–3 months, and none developed long-term adrenal insufficiency.

Predicting who will develop postoperative adrenal suppression-and how long it will last-remains difficult. Morelli et al., in a cohort of patients with adrenal incidentalomas that also included patients with MACS, observed suppression of the hypothalamic–pituitary–adrenal axis in 46% of cases (139). Although this was less frequent than in overt Cushing’s syndrome (82%), it was still significant. Preoperative cortisol levels after DST had limited predictive value, and even very low levels did not reliably rule out postoperative dysfunction. In some patients, recovery was prolonged: suppression lasted more than 18 months in about one-third of cases, and in rare instances exceeded 3 years. While the exact duration could not be predicted, patients with multiple preoperative abnormalities-such as low ACTH, elevated urinary free cortisol, and post-DST cortisol ≥3 μg/dL-were more likely to experience longer recovery. A recent study by Bonaventura et al. in a cohort of 32 non-aldosterone-secreting adrenal adenomas, including 25 MACS cases, showed that early postoperative adrenal suppression is common after adrenalectomy, with only 18.8% of patients having normal function at day 6. However, over half recovered within 6 weeks. Preoperative cortisol after the 1 mg DST was the only significant predictor of early recovery, with lower values associated with faster normalization (15).

Taken together, there is a clear need for more uniform diagnostic criteria and follow-up strategies to avoid unnecessary steroid treatment and better guide patient management.

### 4.2 Pharmacological therapy

Although adrenalectomy remains the preferred treatment for selected patients with unilateral MACS and clinically relevant cortisol-related comorbidities, pharmacological therapy for MACS is not standardized internationally. Availability of steroidogenesis inhibitors and glucocorticoid receptor antagonists differs substantially by country because regulatory approvals generally concern overt endogenous Cushing’s syndrome rather than MACS. In many settings, use for MACS is off-label, limited to clinical trials, or country-specific access programs. This variation should be considered when translating trial results into routine practice. An important clinical consideration regarding these drugs is the potential risk of treatment-induced adrenal insufficiency; however, recent trials using low-dose evening regimens of steroidogenesis inhibitors reported no such episodes (140–143). In Poland, steroidogenesis inhibitors are not readily available for outpatient use.

#### 4.2.1 Steroidogenesis inhibitors

Metyrapone and osilodrostat are orally administered 11β-hydroxylase inhibitors. Metyrapone was initially used diagnostically to assess the hypothalamic-pituitary-adrenal axis, while osilodrostat was approved for the treatment of endogenous CS. A detailed summary of published studies evaluating the cardiometabolic efficacy of metyrapone in MACS, as well as currently ongoing clinical trials evaluating both agents in this setting, is presented in **Table 7**.

**Table 7:** Published and ongoing clinical trials evaluating steroidogenesis inhibitors (metyrapone and osilodrostat) in patients with MACS.

Study	Study design	Sample size/population	Key findings
Published studies			
Debono et al., 2017 (140)	Phase 1/2a prospective study. Intervention: single-day metyrapone 500 mg at 6 pm + 250 mg at 10 pm.	6 MACS; 12 controls (6 NFAT, 6 healthy)	Cortisol rhythm normalized to physiological concentrations (AUC 6 am-10 pm, p=0.85 vs controls, AUC 10 pm-2 am, p=0.76 vs controls). No adrenal insufficiency.
Niziolek et al., 2026 (141)	Prospective open-label trial; 12 weeks. Intervention: evening metyrapone 500 mg at 6 pm + 250 mg at 10 pm.	n=15 MACS	Intrahepatic lipid content decreased from 3.85% to 1.92% (p=0.010, mean relative reduction 27%). Fasting insulin decreased from 12.6 to 9.3 µIU/mL (p=0.041). BP decreased nonsignificantly: SBP 128 → 122 mmHg (p=0.075), DBP 83 → 78 mmHg (p=0.10). No adrenal insufficiency.
Berry et al., 2026 (142)	Retrospective real-world study; 6 months. Intervention: evening metyrapone 250–500 mg at 6 pm + 250 mg at 10 pm.	15 MACS; 15 matched controls	Significant reduction in mean SBP (-17.7 vs +8.7 mmHg in controls, p=0.008) and DBP (-9.9 vs +3.0 mmHg, p=0.024). No significant improvements in HbA1c, weight, or non-HDL cholesterol. No adrenal crises, 2/15 patients discontinued due to side effects.
Musolino et al., 2025 (143)	Prospective observational study; 24 weeks. Intervention: low-dose evening metyrapone 250–500 mg/day.	n=20 MACS.	Clinically significant reduction in 24h SBP (≥ 5 mmHg) in 40% (8/20) of patients without antihypertensive therapy escalation (p=0.008). Changes in DBP were smaller and non-significant. No significant changes in fasting glucose (p=0.45), insulin/glucose AUC, weight (p=0.67), BMI (p=0.74), or UFC (p=0.67). No adrenal insufficiency.
Ongoing trials			
Study	Study design	Purpose of the study	
SUN448, NCT06106295	Phase 2 trial (Mayo Clinic). Intervention: metyrapone initiated at 250 mg nightly with titration up to 1000 mg.	Evaluating safety and tolerability. No cases of adrenal insufficiency have been reported to date.	
NCT07138274	Phase 2 double-blind, placebo-controlled trial (Mayo Clinic). Intervention: metyrapone.	Evaluating safety and efficacy.	
NCT07104812	Phase 2 single-arm trial (Mayo Clinic). Intervention: very low-dose osilodrostat (1 mg daily).	Evaluating safety and tolerability, along with 24h urinary steroid metabolomics and circadian cortisol patterns.	
OSI-CORT	Clinical trial (NIHR Birmingham). Intervention: low-dose osilodrostat.	Exploring steroid metabolism, immunometabolic parameters, and muscle function.	
NCT07247058	Phase 4 clinical trial (Johns Hopkins University). Intervention: osilodrostat.	Evaluating metabolic parameters and quality of life.	
NCT07268222	Phase 4 multicenter interventional trial (University of Athens). Interventions: metyrapone 250 mg, osilodrostat 1 mg, adrenalectomy, or conservative management.	Comparing metyrapone, osilodrostat, adrenalectomy, and conservative management in MACS.	

AUC, area under the curve; BMI, body mass index; BP, blood pressure; DBP, diastolic blood pressure; HbA1c, glycated hemoglobin; HDL, high-density lipoprotein; MACS, mild autonomous cortisol secretion; NFAT, non-functioning adrenal tumors; NIHR, National Institute for Health and Care Research; SBP, systolic blood pressure; UFC, urinary free cortisol.

Access to steroidogenesis inhibitors and other pharmacological therapies for MACS varies considerably across countries or even regions of the same country, reflecting differences in the functioning of healthcare systems, regulatory approvals, reimbursement policies, insurance coverage, and local treatment pathways. Poland illustrates these broader geographic disparities: outpatient access to steroidogenesis inhibitors is limited, metyrapone is generally restricted to inpatient treatment pathways, whereas osilodrostat may require use of the Emergency Access to Drug Technologies mechanism.

#### 4.2.2 Glucocorticoid receptor antagonists

Glucocorticoid receptor antagonists-mifepristone and relacorilant-may be considered as an alternative to steroidogenesis inhibitors in MACS, as they block cortisol action at the tissue level without directly suppressing adrenal cortisol synthesis. So far, the evidence on the feasibility of this approach in MACS is limited and comes from pilot studies and indirect data extracted from larger studies.

A small open‑label study in 8 patients with MACS treated with mifepristone 300 mg daily demonstrated improvements in glucose metabolism and insulin resistance during short‑term mifepristone therapy, suggesting potential metabolic benefit (144). In addition, the recently published phase 4 CATALYST trial, although not designed for MACS, showed that mifepristone significantly improved glycaemic control and cardiometabolic parameters in patients with difficult‑to‑control type 2 diabetes and biochemical evidence of endogenous hypercortisolism identified in a substantial proportion (24%) of screened patients (145, 146). The main adverse effects of this approach include symptoms of glucocorticoid withdrawal and difficulty in monitoring efficacy, as cortisol levels may be elevated during receptor blockade, making monitoring primarily symptom-based. Additional concerns include hypokalaemia, interference with thyroid hormone measurements, and, because mifepristone also has progesterone receptor antagonism, endometrial thickening and vaginal bleeding. By contrast, relacorilant is a glucocorticoid receptor modulator without progesterone receptor antagonism. To date, it has only been studied in overt endogenous Cushing’s syndrome. In a multicenter, open‑label study (NCT02804750) of 35 adults with endogenous CS, it was administered for 12–16 weeks at low (100–200 mg/day) or higher doses (250–400 mg/day, titrated in 50 mg increments every 4 weeks). Improvements in blood pressure were observed in 41.7% of patients receiving low-dose therapy and 63.6% of those receiving high-dose therapy, while improvements in glycaemic control occurred in 15.4% and 50% of patients, respectively (147, 148). Reported adverse events were mild to moderate and included headaches, peripheral edema, nausea, and dizziness, without vaginal bleeding or hypokalaemia.

#### 4.2.3 Other approaches

Beyond steroid‑targeting approaches, metabolic strategies targeting obesity and insulin resistance are being discussed as complementary approaches for patients with MACS, because metabolic complications are frequent in this population. The attribution of the clinical phenotype to MACS remains challenging, particularly in individuals with additional metabolic syndrome risk factors. At the same time, growing evidence underscores the wide-ranging and pleiotropic cardiometabolic benefits of emerging glucagon-like peptide-1 (GLP-1) and dual GLP-1/glucose-dependent insulinotropic polypeptide (GIP)–based therapies. The ongoing phase 4 IMPACT-MACS trial (NCT07361874) is a single-center, prospective interventional study comparing unilateral adrenalectomy with treatment using the GLP-1 receptor agonist semaglutide in adults with MACS. The trial aims to determine whether improvement in insulin resistance, assessed by the hyperinsulinaemic-euglycaemic clamp, is greater following adrenalectomy or after semaglutide. Secondary outcomes encompass a detailed assessment of cortisol dynamics, urinary steroid metabolomics, body composition, blood pressure, lipid profile, inflammatory markers, muscle strength, and quality of life. Additionally, a matched semaglutide-treated control group (without adrenal tumors) with comparable cardiometabolic risk will evaluate whether patients with MACS demonstrate a differential metabolic response to GLP-1 receptor agonism. The results of this study are anticipated to provide important mechanistic insights into the relative contributions of cortisol excess versus obesity‑related pathways to cardiometabolic risk in MACS.

## 5 Practical clinical algorithm

In summary, the clinical management of patients with MACS can be structured around a simple five-step practical algorithm:

1. characterize the adrenal lesion radiologically and exclude other clinically relevant adrenal hormone excess when indicated;
2. perform the 1 mg overnight DST, review confounders, and confirm ACTH independence when post DST cortisol is >50 nmol/L;
3. assess potentially cortisol-related comorbidities, including hypertension, type 2 diabetes or prediabetes, dyslipidemia, osteoporosis/vertebral fractures, obesity, muscle impairment, and relevant neuropsychiatric or functional symptoms;
4. for unilateral MACS with persistent cortisol autonomy and clinically important or progressive comorbidities, discuss adrenalectomy in a multidisciplinary setting, incorporating age, general health, tumor characteristics, expected benefit, and patient preference; for bilateral disease or patients not undergoing surgery, optimize comorbidity treatment and consider specialist follow-up, with pharmacotherapy currently reserved for selected patients and research or expert-center settings;
5. after adrenalectomy, provide perioperative glucocorticoid coverage and reassess HPA-axis recovery, while monitoring the trajectory of the comorbidities that motivated intervention.

## 6 Conclusions

This review aimed to emphasize the complex, multisystem nature of MACS, a syndrome that, in light of current evidence, appears mild only by name. Despite the absence of the classical phenotype of CS and the rarity of progression to overt hypercortisolism, MACS is clearly associated with a broad spectrum of systemic consequences, including cardiovascular disease, hypertension, glucose metabolism disturbances, dyslipidemia, MASLD, skeletal fragility, frailty, sleep disturbances, and neuropsychiatric complaints. These complications translate into increased morbidity and mortality and support the need for practical clinical algorithms for this growing patient population. The main unresolved clinical and research challenges in MACS have been summarized in **Figure 3**. First, current diagnostic strategies remain imperfect. The 1 mg overnight DST is the cornerstone of diagnosis, but its interpretation is limited by false-positive results, the lack of universally accepted risk thresholds, and limited ability to predict clinically relevant outcomes. Additional markers, including both generally recognized such as ACTH and DHEA-S; or novel/emerging such as hair cortisol/cortisone, late-night salivary cortisone, urinary steroid metabolomics, circadian metabolomic signatures, and inflammatory indices, may improve diagnostic precision in the future In this context, artificial intelligence and machine-learning approaches may support the development of predictive algorithms. As not all patients with MACS carry the same clinical risk or derive the same benefit from active intervention, future algorithms should integrate biochemical cortisol secretion, comorbidity burden, age, sex, tumor characteristics, peripheral glucocorticoid metabolism, tissue sensitivity to glucocorticoids, and patient preference. Large collaborative initiatives using retrospective and prospective datasets are underway. Treatment should therefore be individualized. At present, pharmacological treatment is emerging but remains non-standardized. Steroidogenesis inhibitors and glucocorticoid receptor antagonists may represent future alternatives for selected patients, particularly those with bilateral disease or contraindications to surgery. However, evidence is still limited, long-term safety data are lacking, and access is restricted in several countries, including Poland. Metabolic approaches, such as GLP-1 receptor agonist-based therapies, may also help clarify the relative contribution of cortisol excess versus obesity-related mechanisms to cardiometabolic risk.

**Figure 3:**
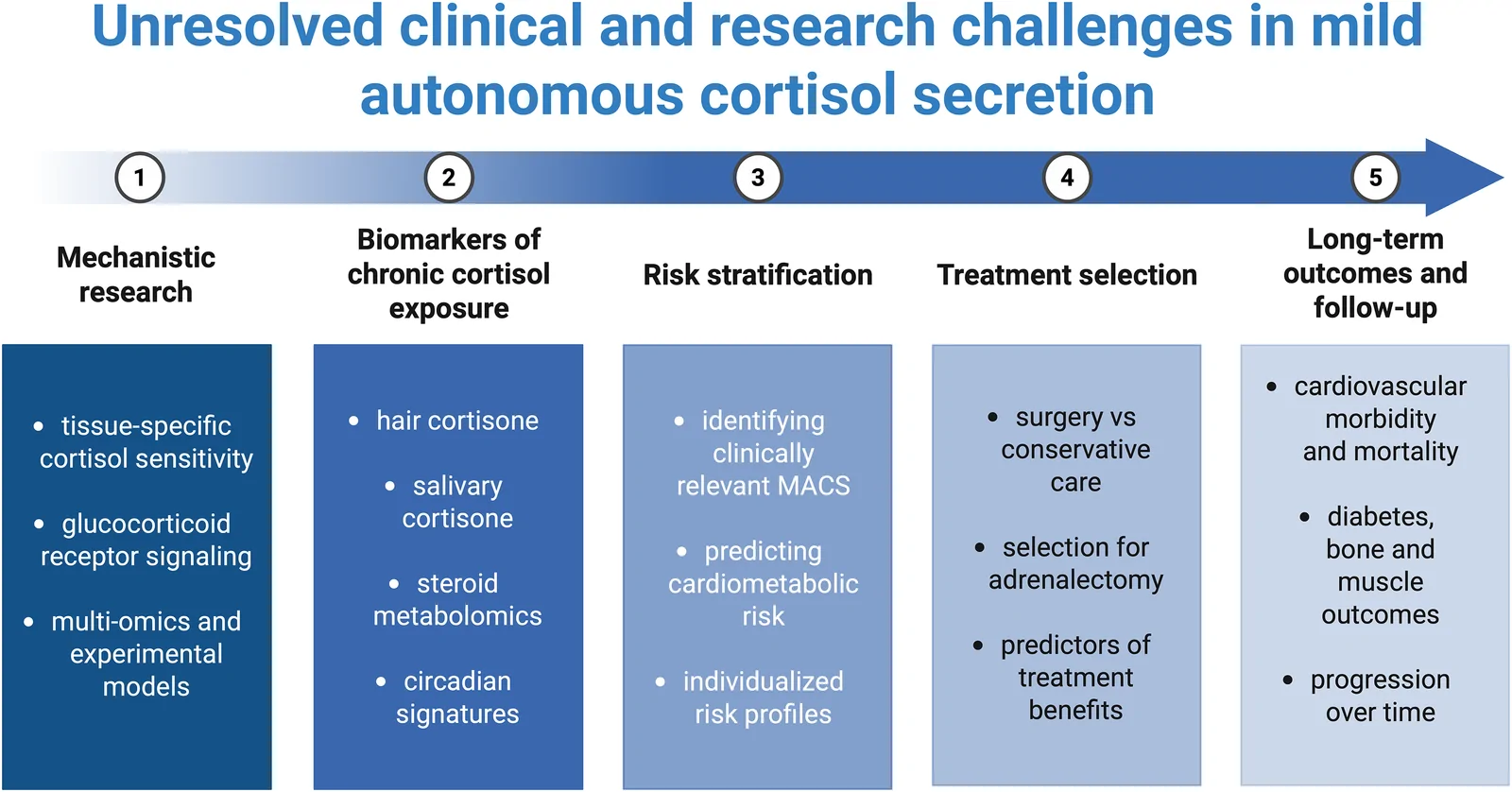
Conceptual framework highlighting the transition from current clinical uncertainty toward more personalized and mechanism-based management of patients with MACS. DST, dexamethasone suppression test; ACTH, adrenocorticotropic hormone; MACS, mild autonomous cortisol secretion.

Overall, MACS should be approached as a heterogeneous, multisystem condition requiring structured assessment and personalized management. Recognizing MACS as a clinically meaningful disorder rather than a borderline biochemical finding is essential for improving long-term outcomes in patients with adrenal incidentalomas. Future research should focus on validated risk prediction models, standardized diagnostic pathways, long-term outcomes after different treatment strategies, prevention of skeletal and cardiovascular complications, and mechanistic studies of tissue-specific glucocorticoid action.
